# A Culturally Grounded Music-Based Intervention for Indigenous Older Adults in Long-Term Care: Protocol for a Mixed Methods Pilot Study

**DOI:** 10.2196/104850

**Published:** 2026-09-01

**Authors:** Ariel L Roddy, Katherine Mommaerts, Rebecca Maniglia, Juliette Roddy, Craig Yarbrough, Julie A Baldwin

**Affiliations:** 1 Northern Arizona University, 5 E Mcconnell Dr., Flagstaff, AZ, 86001, United States, 1 2482986978

**Keywords:** Indigenous health, music-based intervention, study protocol, mixed methods, culturally grounded, mental health

## Abstract

**Background:**

Indigenous communities continue to experience significant mental and physical health disparities associated with historical trauma, structural inequities, and disruptions to cultural continuity. Indigenous older adults residing in long-term care settings may be particularly vulnerable to social isolation, psychological distress, chronic pain, and reduced opportunities for cultural engagement. Music-based interventions (MBIs) have demonstrated potential for improving psychosocial and physical health outcomes; however, few MBIs have been culturally grounded in Indigenous worldviews or codeveloped with Indigenous communities.

**Objective:**

This manuscript reports the protocol for an ongoing pilot feasibility study evaluating a culturally grounded MBI for Indigenous older adults residing in long-term care settings in northern Arizona. The primary objectives are to assess the feasibility, acceptability, and implementation of the intervention, and to generate preliminary data regarding changes in mental health, pain, coping, and cultural connectedness.

**Methods:**

The study uses a quasi-experimental convergent parallel mixed methods design informed by the Indigenist Stress-Coping Model. The intervention was codeveloped through collaboration with a Community Advisory Board consisting of Indigenous elders, clinicians, researchers, and Indigenous musical consultants. The 6-session curriculum incorporates active music participation, including drumming, singing, storytelling, rhythmic engagement, and guided reflection. Participants are recruited from 2 residential long-term care facilities. Quantitative measures are collected before and after intervention participation and include assessments of depression, anxiety, perceived stress, pain, coping, and cultural connectedness. Qualitative interviews, observational field notes, and implementation data are collected concurrently to assess participant experiences, cultural relevance, intervention fidelity, and contextual factors influencing implementation.

**Results:**

This study was first funded in February 2024 by The NARBHA Institute and the James Wurgler, MD Endowed Chair. Continued funding was secured in September 2025 through the National Institute on Minority Health and Health Disparities through the Southwest Health Equity Research Collaborative Pilot Project Program (grant U54MD012388). Recruitment and intervention implementation began in March 2024 and are expected to conclude in May 2027. As of June 2026, 39 participants have been enrolled across 2 residential long-term care facilities. Quantitative and qualitative analyses are expected to begin in summer 2026. Findings will be disseminated through community stakeholder meetings, conference presentations, and peer-reviewed publications, with initial results anticipated in summer 2027.

**Conclusions:**

This protocol describes the development and evaluation of a culturally grounded MBI designed to support mental, physical, and cultural well-being among Indigenous older adults. Findings will inform intervention refinement, feasibility assessment, and future large-scale studies evaluating culturally responsive behavioral health interventions for Indigenous communities.

## Introduction

### Background

Indigenous communities in the United States continue to experience significant health disparities driven by complex intersections of historical trauma, structural inequities, and cultural disruption. These disparities are particularly acute in mental and physical health outcomes, including elevated rates of depression, anxiety, stress, and chronic pain, especially among Indigenous elders [1]. The COVID-19 pandemic magnified these inequities, with Indigenous populations experiencing the highest mortality rates nationwide [2]. These losses disproportionately affected tribal elders, many of whom were critical cultural knowledge bearers, thereby intensifying experiences of spiritual grief, cultural loss, and psychological distress within communities [3].

Cultural protective factors, including traditional music, ceremony, and community cohesion, have long played a central role in fostering resilience among Indigenous peoples [4-6]. Cultural mentors and traditional modalities in arts-based frameworks have increasingly demonstrated success in fostering an enhanced sense of cultural identity and safe self-expression within Indigenous settings [7]. However, few behavioral health interventions have meaningfully integrated these cultural strengths; most existing interventions are either inadequately tailored or designed without Indigenous input, resulting in low engagement and limited efficacy across diverse tribal contexts [8]. In particular, music-based interventions (MBIs) have demonstrated potential to alleviate symptoms of depression, anxiety, and physical pain [9,10], yet existing literature rarely considers the cultural relevance or contextual specificity of the musical content. The unique healing properties of music, especially when grounded in cultural traditions, remain underexplored in Indigenous health research [5,11,12].

### Behavioral Interventions, Music, and Health

MBIs are an emerging form of behavioral health treatment that harness the therapeutic effects of music on emotion regulation, stress reduction, and physical symptom relief [13,14]. Studies across clinical and community-based settings show that music can reduce perceived pain, lower stress biomarkers, and support psychological healing [15,16]. Furthermore, studies show that drumming has been shown to lower biological stress markers, providing a pathway for managing chronic, trauma-related stress [17]. In chronic care contexts, music has been found to significantly reduce anxiety and depressive symptoms while also promoting social interaction, which is especially critical for isolated or institutionalized populations [18,19]. MBIs vary in form, from structured music therapy led by clinicians to informal group drumming and singing, but active music participation—such as creating rhythms, singing, or moving to music—has been shown to produce more profound psychological effects than passive listening alone [20].

Despite this promise, most MBIs lack cultural specificity or fail to adapt to the social realities of marginalized communities [21]. Cultural context plays a significant role in how individuals engage with music, and a one-size-fits-all model may fall short when applied in settings such as tribal nations, where music traditions are deeply rooted in ceremonial, relational, and intergenerational practices.

### Culturally Grounded MBIs for Indigenous Communities

While traditional music, ceremony, and storytelling are well-established cultural resources within Indigenous communities, these elements are rarely integrated into mainstream behavioral interventions. Colonial legacies have created long-standing barriers to incorporating Indigenous healing practices into clinical models, contributing to underrepresentation and mistrust in behavioral health systems [22]. Indigenous paradigms view health through a holistic lens of social and emotional well-being—one that connects individual wellness directly to community vitality, ancestral roots, and the land, thereby moving beyond the more segmented definitions of Western medicine [23]. Yet, traditional music holds profound potential as a healing modality—it is often used to mark transitions, connect with ancestors, build community, and restore emotional balance.

A culturally grounded MBI, co-designed with Indigenous stakeholders, offers a powerful strategy for addressing disparities while honoring local knowledge. Integrating tribal-specific traditions such as drumming, flute playing, or call-and-response singing ensures that the intervention resonates with participants’ lived experiences. Furthermore, the codevelopment process itself can serve as a form of community healing by affirming the value of Indigenous knowledge and fostering collaborative leadership.

This study builds on practice-based evidence within Indigenous communities, transforming it into a structured, evaluable curriculum that can be adapted across settings. In doing so, we seek not only to improve outcomes for Indigenous elders in long-term care settings but also to create an evidence-informed model for other cultural groups underrepresented in behavioral health research.

### Theoretical Framework: The Indigenist Stress-Coping Model

The Indigenist Stress-Coping Model (ISCM) provides the conceptual foundation for this project. Developed by Walters and Simoni [24], the ISCM emphasizes the protective role of cultural identity, traditional practices, and social support in buffering the effects of trauma. This framework is particularly relevant for populations experiencing both historical and ongoing systemic oppression, including Native communities across the United States, and has been used in other contexts to provide a structural framework through which to develop culturally relevant interventions [25,26].

Within the ISCM, health disparities are seen as rooted not only in individual behavior but also in collective histories of forced assimilation, land loss, and intergenerational trauma. Music—as a cultural, relational, and spiritual practice—offers a powerful avenue for reclaiming Indigenous identity and promoting resilience. Participation in culturally meaningful musical activities may counteract cultural isolation, restore community ties, and reduce the psychological and physical toll of accumulated stress. Integrating cultural strengths into research designs requires an ethical approach that respects traditional values while still showing that cultural practices and beliefs serve as measurable protective factors against structural stress [27].

**Figure 1.**
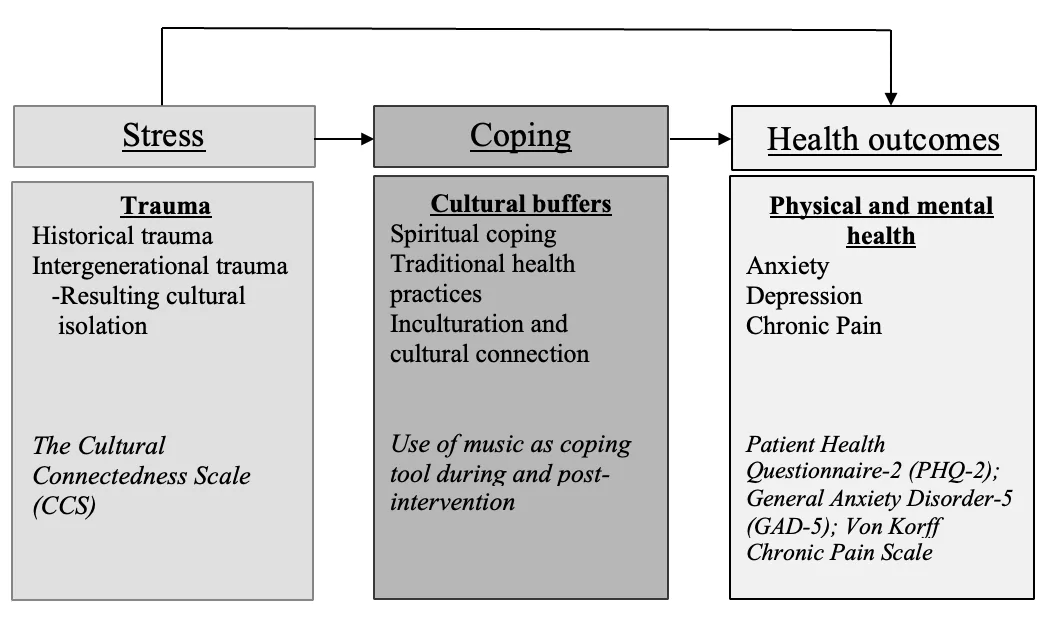
Conceptual framework illustrating application of the Indigenist Stress-Coping Model [24] to the culturally grounded music-based intervention evaluated in an ongoing pilot feasibility study among Indigenous older adults residing in residential long-term care settings.

In this study, we apply the ISCM to understand how music participation can improve health outcomes, particularly among elders who may feel disconnected from traditional practices. We hypothesize that culturally grounded music engagement can serve as both a coping mechanism and a form of resistance, offering healing in the face of historical adversity. Figure 1 provides an outline of the relevant relationships outlined in the ISCM as they pertain to the current study.

### Current Study

To address gaps in culturally responsive behavioral health interventions for Indigenous older adults, we are conducting an ongoing pilot feasibility study of a culturally grounded MBI developed through a community-engaged process. The intervention was codeveloped in partnership with a Community Advisory Board (CAB) composed of Indigenous elders, musical consultants, clinicians, researchers, and community stakeholders. This collaborative approach ensures that the intervention reflects Indigenous values, cultural traditions, and community priorities while remaining adaptable across multiple tribal contexts.

The intervention is currently being implemented across long-term care facilities in northern Arizona. Because participating residents represent diverse tribal affiliations and varying levels of cultural connectedness, the curriculum was intentionally designed to balance cultural specificity with cross-tribal applicability. Indigenous musical traditions, including drumming, flute music, storytelling, and collective music-making, serve as central mechanisms for promoting cultural engagement, social connection, and emotional well-being.

Guided by the ISCM [24], the intervention is conceptualized as a culturally grounded coping resource that may mitigate the effects of cultural isolation, historical trauma, and psychosocial stress. Specifically, participation in culturally meaningful musical activities is hypothesized to strengthen cultural connectedness, enhance coping capacity, promote social engagement, and ultimately improve mental and physical health outcomes.

The current manuscript reports the protocol for this ongoing pilot feasibility study. The primary goals are to evaluate feasibility, acceptability, fidelity, and implementation processes while generating preliminary data to inform future efficacy testing.

### Specific Aims of the Study

The study has 2 main objectives:

- Aim 1: Modify and refine a culturally and contextually appropriate MBI curriculum with iterative input from a CAB.
- Aim 2: Assess the preliminary impact of the MBI through a quasi-experimental design incorporating both quantitative and qualitative data.
  - Aim 2a: Assess changes in the use of music as a coping strategy for mental and physical health.
  - Aim 2b: Evaluate changes in mental (anxiety, depression, perceived stress) and physical health symptoms (pain) following participation in the MBI.
  - Aim 2c: Compare outcomes across subgroups (Indigenous vs non-Indigenous; culturally isolated vs culturally connected).

In this paper, we describe the study design, curriculum development, implementation procedures, and evaluation strategies used to assess outcomes across participants, Indigenous musicians, and community facilitators.

## Methods

### Design

We will conduct a quasi-experimental, mixed methods study from the perspectives of participants, Indigenous musicians, and community health group facilitators in Arizona from fall 2024 through 2027. The study design integrates quantitative and qualitative data to examine the feasibility, acceptability, and perceived impacts of a culturally grounded MBI delivered in community settings. Multiple stakeholder perspectives are included to capture both individual-level experiences and implementation-related factors relevant to community-based health programming.

The CAB will guide the design and refinement of the MBI (Aim 1). The CAB includes experts in music pedagogy and curriculum development, Indigenous community members with cultural and musical expertise, and practitioners familiar with the study populations. The CAB meets at 3 points during each curriculum cycle: curriculum development, content review, and review of initial findings following delivery. CAB members support the research team in developing and piloting an MBI curriculum for use with Indigenous and non-Native populations in residential and community-based settings.

Research team members, including Indigenous investigators, have developed a 6-session scripted curriculum grounded in the Indigenous Medicine Wheel [28], which aligns the 4 cardinal directions with emotional, physical, mental, and spiritual dimensions of human experience. Each session begins with an introduction by the Indigenous musical consultant describing the Medicine Wheel, the session’s geographic direction, and its associated dimension. Each session includes two components: (1) an interactive MBI facilitated by a trained facilitator (eg, case manager or licensed mental health therapist), and (2) a drumming and flute session led by an Indigenous music consultant. Consultants are compensated and selected to represent diverse Indigenous backgrounds and expertise. All facilitators and consultants receive standardized training in the curriculum as well as written curriculum materials to ensure consistent delivery and shared understanding of the Indigenous foundations of the intervention.

To support fidelity, the curriculum is scripted and delivered using a consistent structure. Participants are greeted individually by the Indigenous musical consultant and offered optional smudging. Sessions incorporate guided reflection, group discussion, and participatory music-making using Indigenous instruments. Each session concludes with a culturally grounded closing led by the musical consultant.

Following the initial delivery, feedback is collected from participants, facilitators, and Indigenous music consultants. Curriculum adaptations are reviewed with the CAB, incorporated as appropriate, and addressed through additional training prior to subsequent delivery cycles.

To assess the preliminary impact of the culturally appropriate MBI (Aim 2), we employ a quasi-experimental, dose-response, pretest-posttest design embedded within a convergent parallel mixed methods framework [29]. This design allows for simultaneous collection and integration of quantitative and qualitative data to assess individual-level change, while also capturing contextual and experiential insights from participants, facilitators, and caregivers. The dose-response model is particularly well-suited to this intervention, as it enables examination of the relationship between treatment exposure (ie, number of sessions attended and type of participation—active vs passive) and changes in mental health, physical health, and psychosocial outcomes.

A quasi-experimental approach is appropriate at this pilot and feasibility stage, particularly in a community-based setting with vulnerable populations where randomized controlled trials may be infeasible or ethically inappropriate. Given that the study population includes older adults, some with cognitive impairment or complex care needs, randomization and withholding a potentially beneficial intervention would pose ethical challenges. Moreover, the intervention is grounded in community participation and cultural traditions, which makes standardization across randomized groups less desirable in this early phase. Instead, the dose-response design allows for rigorous analysis of preliminary effects, while respecting ethical considerations and cultural priorities. Using collaborative leadership and truth-telling helps build basic trust, honors tribal elders’ lived experience, and addresses barriers that keep people from trusting behavioral health systems [30].

The study activities have been approved by the university’s institutional review board (IRB protocol number 2185298), and any requested modifications in the protocol will be addressed and approved.

### Study Setting

This study is conducted in northern Arizona, a region characterized by substantial geographic dispersion, limited health care access in rural areas, and a high proportion of Indigenous populations. The area includes multiple Tribal Nations and communities where cultural traditions, including music, play a central role in community life and well-being. These regional characteristics shape both access to care and the types of culturally relevant interventions that may be most effective.

The intervention is implemented across 2 residential long-term care facilities that differ in their demographic composition and community context. One site primarily serves Indigenous residents, with approximately 70% of the population identifying as Indigenous, reflecting the surrounding Tribal communities. The second site serves a more heterogeneous population, with an estimated 5% to 10% of residents identifying as Indigenous. These differences provided an opportunity to examine the implementation of a culturally grounded intervention across settings with varying levels of cultural representation and community connectedness.

Both facilities provide skilled nursing and long-term care services to older adults with a range of physical, cognitive, and behavioral health needs. The inclusion of 2 distinct sites enhances the ecological validity of the study and allows for consideration of how cultural context and population composition may influence engagement with and response to the MBI.

### Participants and Recruitment

As noted previously, participants are recruited from 2 residential long-term care and skilled nursing facilities located in northern Arizona. These sites serve a demographically diverse population, including a substantial proportion of Indigenous older adults. Eligibility criteria include (1) current residency at one of the participating care facilities and (2) ability to communicate in English. For participants with diminished decision-making capacity due to cognitive impairment, proxy consent is obtained from legally authorized representatives in accordance with IRB protocols.

Recruitment is conducted in partnership with facility staff, who assist in identifying eligible participants. Outreach materials, such as flyers, in-person presentations, and informational sessions, were developed with guidance from the CAB to ensure cultural relevance and accessibility for elders and their families. All participants are recruited primarily through facility-based outreach and collaboration with staff, with supplementary recruitment through community partnerships as appropriate. The CAB, composed of Indigenous musical consultants, community leaders, clinicians, and researchers, has reviewed and advised on all recruitment strategies. CAB members are compensated for their participation. As this is a pilot feasibility study, a formal power calculation was not conducted. The target sample size is based on feasibility considerations, including site capacity and anticipated recruitment within the study timeframe.

### Intervention Curriculum

The MBI consists of 6 weekly, 60-minute group sessions facilitated by an Indigenous musician and a trained facilitator. The curriculum is developed through a community-engaged process and refined with input from a CAB composed of Indigenous elders, musicians, clinicians, and community stakeholders. The intervention emphasizes active participation in music-making, including drumming, rhythm exercises, movement, singing, and engagement with Indigenous instruments such as the Native flute. Sessions are organized around themes aligned with the dimensions of the Medicine Wheel, including physical relaxation, emotional expression, empowerment, coping, and community connection. Each session follows a standardized structure consisting of a culturally appropriate welcome, session introduction, music-based activity, drumming and instrument engagement, guided reflection, and closing. Cultural teachings related to Indigenous understandings of music, healing, community, and the sacred role of the drum are integrated throughout the curriculum. While the curriculum maintains a consistent structure across sites, facilitators are encouraged to adapt activities as needed to accommodate participants’ physical abilities, cognitive functioning, and group dynamics while preserving the core intervention components. Table 1 outlines the curriculum, including session names, relevant themes, the primary activities of each session, relevant cultural components, and intended outcomes.

**Table 1.** Overview of the 6-session culturally grounded music-based intervention curriculum implemented as part of an ongoing pilot feasibility study among Indigenous and non-Indigenous older adults residing in residential long-term care facilities^a^.

Session	Theme	Primary activities	Cultural components	Intended outcomes
Week 1: “Music as Relaxation”	Exploring music as a tool for physical relaxation and stress reduction	Guided relaxation exercise accompanied by live drumming and flute music; introduction to instruments; group reflection	Introduction to the Medicine Wheel; discussion of the drum as the heartbeat of Mother Earth; Indigenous perspectives on healing through music	Reduce tension, promote relaxation, and increase awareness of music as a calming strategy
Week 2: “External and Internal Control”	Music, empowerment, and collective strength	Group singing; individual and group drumming; call-and-response exercises; pow wow drumming	Medicine Wheel emotional dimension; discussion of collective strength, community, and Indigenous drumming traditions	Enhance feelings of agency, empowerment, emotional regulation, and social connection
Week 3: “Draw What You Hear”	Music, emotion, and memory	Listening to diverse musical styles; discussion of emotional responses and memories; group drumming activities	Indigenous flute and drum music; reflection on ancestral connections and emotional experiences through music	Increase emotional awareness, memory recall, self-expression, and social engagement
Week 4: “Using the Body to Make Music”	Physical engagement and music creation	Body percussion exercises; movement to live music; individual drumming activities	Medicine Wheel physical dimension; Indigenous perspectives on dance, movement, and embodied music-making	Promote physical activity, body awareness, participation, and engagement
Week 5: “Musical Coping”	Music as a coping strategy	Instrument play focused on emotional expression; discussion of coping with stress, sadness, and anger; group music-making	Medicine Wheel mental dimension; discussion of music and ceremony as traditional coping and healing practices	Strengthen coping skills, emotional expression, resilience, and self-regulation
Week 6: “Wrap-Up and Final Exploration”	Reflection and participant-directed music engagement	Participant-selected instruments and activities; open music session; group discussion and reflection	Re-engagement with preferred Indigenous musical practices and instruments; collective reflection	Consolidate learning, reinforce social connection, and encourage ongoing use of music for well-being

a Each 60-minute session followed a standardized structure consisting of (1) a culturally appropriate welcome, (2) session overview and introduction, (3) a music-based activity, (4) Indigenous drumming and instrument engagement led by an Indigenous musician, (5) group reflection, and (6) a culturally appropriate closing. Sessions were designed around the 4 dimensions of the Medicine Wheel (physical, emotional, mental, and spiritual) and emphasized active participation, cultural connection, and community engagement.

### Study Measures

Quantitative data are collected at 2 time points: 1 week prior to the first session (week 0) and 1 week following the final session (week 6). The survey battery includes validated instruments to assess mental and physical health symptoms, psychosocial well-being, and cultural connectedness. Depression symptoms are measured using the Patient Health Questionnaire-9, and anxiety is assessed with the Generalized Anxiety Disorder-7 Scale. The Perceived Stress Scale-10 is used to measure perceived stress, and the Von Korff Chronic Pain Scale captures pain intensity and interference.

Participants also complete scales assessing psychosocial outcomes, including a music coping index adapted from previous MBI research, as well as items related to belonging and self-esteem. Cultural identity and isolation are assessed using selected items from the Cultural Connectedness Scale, enabling comparison across levels of cultural affiliation. These quantitative measures are used to address Aim 2a (use of music as a coping strategy), Aim 2b (changes in health symptoms), and Aim 2c (differential outcomes by Indigenous identity and cultural connectedness).

Qualitative data are collected through semistructured interviews conducted within 1 week of the intervention’s conclusion (week 7). These interviews involve a subset of participants, as well as caregivers and facility staff, to explore perceived changes in mental, emotional, and physical well-being; group engagement; and cultural relevance of the intervention. In cases where participants are unable to complete interviews due to cognitive limitations, caregivers serve as informants. Weekly fidelity observations are also conducted by research team members attending the sessions. These field notes document participant engagement, group dynamics, musical interaction, and any notable changes in behavior or affect over time. CAB meeting notes are reviewed to capture contextual insights related to implementation and cultural alignment of the MBI curriculum.

### Data Analysis

Quantitative data will be analyzed using descriptive and inferential statistical methods. Participant characteristics, feasibility outcomes, and implementation metrics will be summarized using frequencies, percentages, means, and standard deviations, as appropriate. Paired-sample, 1-tailed *t* tests will be used to assess within-subject changes from preintervention to postintervention across the primary outcome domains. Effect sizes (Cohen *d*) will be calculated to estimate the magnitude of observed changes [31]. Exploratory dose-response analyses will examine whether intervention dose, defined as the number of sessions attended, is associated with changes in mental health, physical health, coping, pain, and cultural connectedness outcomes using linear regression models. If participant engagement is systematically assessed through observational measures, engagement level will also be explored as a predictor of outcome change. Descriptive subgroup analyses may be conducted by study site, Indigenous identity, and baseline cultural connectedness when sample sizes permit. Because this pilot study is not powered to detect subgroup differences, these analyses will be considered exploratory and interpreted cautiously to inform intervention refinement and the design of future efficacy trials.

To account for missing data, diagnostics are conducted to determine if data are missing completely at random or missing at random. When appropriate, multiple imputation is used to preserve sample size and statistical power. All quantitative analyses are conducted using Stata 18 statistical software.

Qualitative data are analyzed through thematic analysis using an inductive coding approach. Audio-recorded interviews are transcribed verbatim, and analysis proceeds in stages: initial open coding to identify emergent concepts, followed by axial coding to organize themes, and selective coding to refine categories. Coding is conducted using ATLAS.ti 14 software [32]. Two independent coders analyze the data, and discrepancies are resolved through regular team meetings. Session observation notes and CAB documentation are included in the analysis to triangulate findings and improve the depth and validity of interpretations.

In addition, this pilot will specifically report on the feasibility and acceptability of the intervention. The primary outcomes of this pilot study are feasibility and acceptability. Feasibility will be evaluated using recruitment rates, participant retention, intervention attendance, completion of outcome assessments, and intervention fidelity. Feasibility will be considered acceptable if 80% of enrolled participants complete the postintervention assessment and participants attend an average of 4 or more of the 6 intervention sessions. Intervention fidelity will be assessed using standardized observation checklists completed by research staff and will be summarized descriptively.

Acceptability will be evaluated using participant satisfaction ratings, qualitative interview data, and observational field notes. Acceptability will be considered favorable if the majority of participants report positive perceptions of the intervention and qualitative findings indicate that the curriculum was culturally relevant, engaging, and appropriate for implementation within long-term care settings (Table 2).

**Figure 2.**
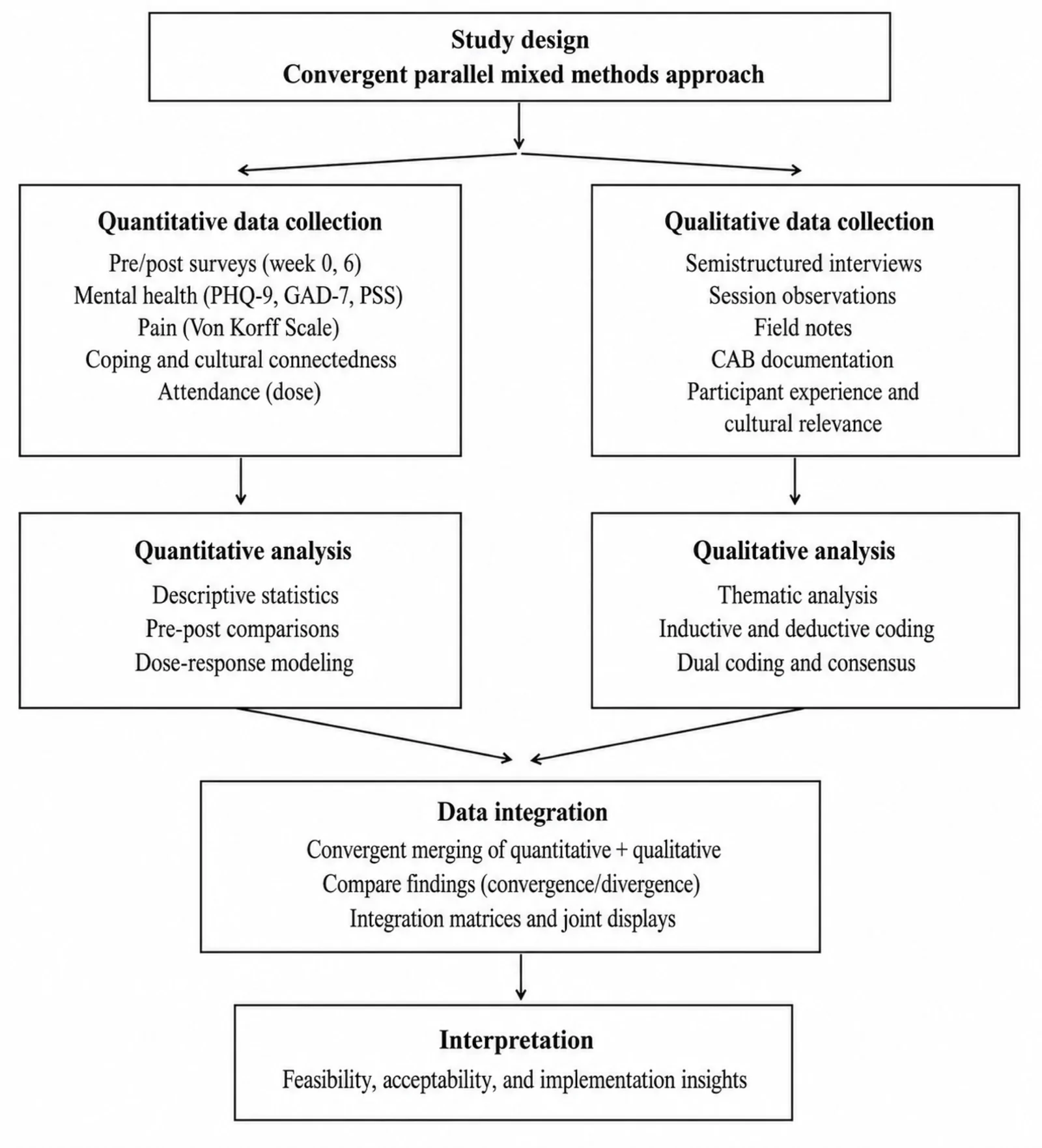
Convergent parallel mixed methods study design used to evaluate the feasibility, acceptability, implementation, and preliminary outcomes of a culturally grounded music-based intervention among older adults residing in residential long-term care facilities. Convergent parallel mixed methods design. Quantitative and qualitative data are collected concurrently, analyzed separately, and integrated during interpretation to assess feasibility, acceptability, and implementation of the music-based intervention. CAB: Community Advisory Board; GAD-7: Generalized Anxiety Disorder-7 Scale; PHQ-9: Patient Health Questionnaire-9; PSS: Perceived Stress Scale.

Integration of quantitative and qualitative data will follow a convergent parallel mixed methods design (see Figure 2). Data strands will be analyzed independently and then merged at the interpretation stage to identify areas of convergence (eg, alignment between self-reported improvement and observed behavioral changes) and divergence (eg, variation in outcomes based on cultural context or engagement style). Integration matrices will be used to systematically synthesize findings across data sources, and representative case examples will be included to illustrate key patterns [29].

**Table 2.** Planned feasibility, acceptability, fidelity, and implementation outcomes and corresponding operational measures for evaluating a culturally grounded music-based intervention among older adults residing in residential long-term care facilities.

Outcome	Definition	Success criterion	Measure
Retention	Participant completion of the 6-week intervention	80% completion of postintervention survey or more	Postintervention surveys
Attendance	Intervention dose (ie, number of sessions completed)	Mean attendance of 4 sessions or more	Attendance records
Fidelity	Delivery as intended	90% of core intervention components delivered	Researcher observation forms
Acceptability	Participant satisfaction	80% positive interview or satisfaction responses	Interviews, observation forms

### Ethical Considerations

This study was approved by the Northern Arizona University IRB (protocol number 2185298), and a University Office of Native American Initiatives was consulted to ensure that the project and recruitment are in alignment with a state and tribal compact that governs the required tribal oversight for research with Native American populations in the state of Arizona. The implementation study is currently registered on ClinicalTrials.gov (identifier: NCT07538427).

Because the study included older adults residing in long-term care settings, including individuals with cognitive impairment, participant capacity to provide informed consent is assessed in collaboration with facility staff. Participants with decisional capacity provide written informed consent before participation. For individuals who lack decisional capacity, written permission is obtained from a legally authorized representative, and participant assent is obtained.

Consistent with the community-engaged nature of this study, the intervention curriculum, recruitment procedures, and implementation strategies were developed and refined in collaboration with a CAB composed of Indigenous elders, clinicians, researchers, and Indigenous musicians, with additional consultation from Northern Arizona University’s Office of Native American Initiatives. Findings will be shared with participating long-term care facilities, CAB members, and other community partners to support knowledge translation, future implementation, and continued community engagement.

## Results

This study was initially funded in February 2024 by The NARBHA Institute and the James Wurgler, MD Endowed Chair to support community engagement, curriculum development, and refinement of the intervention. Continued funding was secured in September 2025 through the National Institute on Minority Health and Health Disparities Southwest Health Equity Research Collaborative Pilot Project Program (grant U54MD012388) to support implementation and evaluation of the intervention described in this protocol. The intervention protocol, study procedures, and statistical analysis plan were finalized prior to participant recruitment for the implementation phase.

Recruitment began in March 2024 and remains active at the time of manuscript submission. Participants continue to be enrolled across both participating long-term care facilities. Data collection is currently underway. Currently, 39 participants have enrolled in the study. The average participant age is 65 years old (mean 64.50, SD 11.94 y). One (2.56%) participant identifies as Black, 3 (7.69%) identify as Hispanic, 16 (41.03%) identify as White, and the remaining participants identify as Indigenous across many different Tribal Nations (n*=*19; 48.72%). Quantitative assessments, qualitative interviews, fidelity observations, and implementation measures are being collected according to the procedures described in this protocol. Outcome analyses have not yet been conducted. Data collection is anticipated to conclude in May 2027, with completed analyses by August 2027.

The anticipated outcomes of this pilot study include estimates of intervention feasibility, acceptability, implementation fidelity, recruitment and retention rates, and preliminary effect size estimates for mental health and pain outcomes, as well as estimates of dose-response relationships. Findings will be disseminated through community stakeholder meetings, conference presentations, and peer-reviewed publications and will be used to refine the intervention curriculum and inform the design of future controlled trials.

## Discussion

### Expected Contributions

This study protocol describes the development and planned evaluation of a culturally grounded MBI designed for Indigenous older adults in long-term care settings. Grounded in the ISCM [24], this work integrates Indigenous knowledge systems with community-engaged research methods to address persistent mental and physical health disparities among Indigenous populations.

This study is expected to contribute to the literature by advancing culturally responsive approaches to behavioral health intervention design and evaluation. Prior research demonstrates the analgesic and psychological benefits of MBIs, including reductions in pain, anxiety, and depression [9,33]. However, the translation of practice-based knowledge from traditional ceremonial practices, such as drumming, into clinical research has been limited due to the enduring impacts of colonization and the historical devaluation of Indigenous knowledge systems [4]. By grounding the intervention in the ISCM, this study applies a culturally relevant framework that situates health disparities within broader contexts of historical and systemic trauma while recognizing Indigenous coping strategies as valid and measurable sources of resilience.

In bridging Indigenous practice-based knowledge with Western research paradigms, this protocol demonstrates how culturally tailored MBIs can be developed in ways that respect Indigenous epistemologies while maintaining methodological rigor. More broadly, this study advances methodological innovation by integrating Indigenous frameworks into intervention research rather than adapting Western models in isolation. Building on prior work, including the DARTNA (Drum-Assisted Recovery Therapy for Native Americans) protocol [11,12], this project customizes the intervention for Southwestern tribal communities while incorporating a dose-response design to examine how varying levels of engagement may influence outcomes. This approach is expected to provide insight into the feasibility, acceptability, and scalability of culturally grounded MBIs across diverse tribal contexts.

### Strengths and Limitations

A central strength of this study lies in balancing cultural relevance with applicability to a multitribal audience. By involving Indigenous musical consultants and a CAB, the study prioritizes community engagement, cultural alignment, and ethical responsiveness while maintaining flexibility across diverse groups. This approach addresses persistent health disparities, particularly among Indigenous older adults, and highlights how interventions can be culturally grounded, community-informed, and scientifically rigorous.

Several anticipated limitations should be considered. First, variability within and across Indigenous communities, including differences in cultural traditions, generational experiences, and levels of cultural connectedness, may influence how participants engage with the intervention. As such, a “one-size-fits-all” approach may not be appropriate, and ongoing adaptation may be necessary to ensure cultural relevance. Second, implementation within long-term care settings may present logistical challenges, including participant attrition, inconsistent attendance, and variability in cognitive and physical functioning. While the study incorporates caregiver input and observational data to address these factors, these challenges may still influence engagement and data completeness. Third, as a quasi-experimental pilot study without a randomized control group, the design may limit the ability to establish causal relationships in future analyses. Additionally, the study’s focus on 2 facilities in northern Arizona and the anticipated sample size may limit generalizability and statistical power, particularly for subgroup analyses.

Practical considerations related to sustainability are also anticipated. Long-term implementation of culturally grounded MBIs will require dedicated funding, trained facilitators, and ongoing capacity-building within community and tribal systems. Continued engagement of CAB members and tribal leaders will be essential to maintaining cultural integrity while supporting adaptation and scalability. Furthermore, limited prior research on MBIs within Indigenous populations underscores the need for continued investigation into how such interventions can be systematically adapted across diverse contexts while maintaining fidelity to both cultural and therapeutic principles.

### Future Directions

Despite these anticipated challenges, this study is designed to generate critical feasibility, acceptability, and implementation data to inform future research. Findings from this pilot are expected to guide the refinement of the intervention curriculum and support the development of larger-scale studies, including randomized or controlled designs. Future research should also examine long-term outcomes and explore underlying mechanisms of change, such as cultural identity, social connection, and spiritual engagement. Additionally, expanding this work to include tribal-specific adaptations and intergenerational programming may further enhance cultural relevance and impact.

Ultimately, this protocol reflects a commitment to community-led and Indigenous-engaged research. By centering Indigenous knowledge systems and prioritizing community partnerships, this study seeks to ensure that interventions are ethically grounded, culturally responsive, and aligned with the needs of the populations they are intended to serve. The long-term goal is to contribute to the reduction of mental and physical health disparities among Indigenous older adults through strengths-based, culturally grounded approaches that promote holistic well-being.

### Conclusion

This protocol describes an ongoing pilot feasibility study evaluating a culturally grounded MBI for Indigenous older adults residing in long-term care settings. By integrating Indigenous knowledge systems, community-engaged research practices, and mixed approaches to promoting methods evaluation, the study seeks to advance culturally responsive approaches to behavioral health intervention development and implementation. The project addresses critical gaps in the literature regarding the use of active MBIs with Indigenous populations and contributes to a growing body of research on culturally relevant, nonpharmacological approaches to promoting health and well-being. Findings from this pilot will inform future intervention refinement, support the development of larger-scale efficacy studies, and provide guidance for implementing culturally grounded health promotion programs in diverse residential care and community settings. Ultimately, this work aims to strengthen the evidence base for leveraging cultural assets and community partnerships to reduce health disparities and improve quality of life among Indigenous and other underserved populations.

## Supplementary material

10.2196/104850 Peer Review Report 1 Peer review report by the National Institute on Minority Health and Health Disparities of the NIH (U54MD012388).

## Abbreviations

CAB: Community Advisory Board
DARTNA: Drum-Assisted Recovery Therapy for Native Americans
IRB: institutional review board
ISCM: Indigenist Stress-Coping Model
MBI: music-based intervention
