# Supplementary material for: A Culturally Grounded Music-Based Intervention for Indigenous Older Adults in Long-Term Care: Protocol for a Mixed Methods Pilot Study
Source: JMIR Res Protoc. 2026 Sep 1;15:e104850. doi: 10.2196/104850 (PMC13532690; doi:10.2196/104850)
Supplement: Supplementary file 1 [file resprot-v15-e104850-s001.pdf]

<u>**SHERC PPP Letter of Intent Scoring Sheet**</u>

Reviewer Name: Reviewer #1
Proposal PI Name: Katie Mommaerts

This scoring sheet will be shared with the applicant.

Strengths and Weaknesses Summary Template
Please provide a short summary (1 – 2 sentences) of strengths/weaknesses in each of the LOI instructions/guidelines categories (a – e) below.

LOI instructions/guidelines	Strengths	Weaknesses
LOI instructions for a – d below are to: *Briefly describe the nature and scope of the project to include the following:*		
a. Goals of the proposed research	- Good, compelling argument as to why this is a problem and that music is an intervention that might work for this group.	
b. Objectives of the research proposed (e.g., to test a stated hypothesis, create a novel design, solve a specific problem, challenge an existing paradigm or clinical practice, address a critical barrier to progress in the field, or develop a new technology)	- You could also use the non-Indigenous group as a control group for the aspect of familiarity contributing to better physical and mental health (unless the sample size isn't large enough to detect an effect OR the control is "contaminated" from being exposed to Navajo culture in Haven).	- Including specific aims here would be really helpful to understand how the design and implementation of the intervention will be broken down and the type of effort that will be needed for each part.<br>- Is music going to be recorded and played or will there be live music? How long is the intervention and how often do they get music?<br>- Is there an existing relationship with Haven Health? Will any Indigenous-specific approvals be needed for the project (again, thinking time)?

c. Summary of the expected outcome, including impact of the results in the research field(s) involved; and	- The expected outcomes address questions that arise in the “Objectives” section.<br>- Clear explanation of the team’s hypotheses and how the results could influence future research.	- It seems like there are a lot of data being collected. Will this amount of data be feasible to collect in the PPP award period?
d. How the objectives align with the goals of SHERC and relevance to health equity. (See definitions of health equities and health disparities written in the LOI guidelines document)	- Great tie to the goals of SHERC.	
e. Investigators		
Is a co-investigator from different area of expertise named? Is the investigative team appropriate?	One PI from social work and one from criminology. One PI is Indigenous. Both PIs have skills that fit together to fulfill the needs of this study.	Could highlight which (or both) investigators’ experience with qualitative feasibility and acceptability data.
**Overall Score (1 – 9): 4**		

Impact	Score	Descriptor	Additional Guidance on Strengths/Weaknesses
High	1	Exceptional	Exceptionally strong with essentially no weaknesses
	2	Outstanding	Extremely strong with negligible weaknesses
	3	Excellent	Very strong with only some minor weaknesses
Medium	4	Very Good	Strong but with numerous minor weaknesses
	5	Good	Strong but with at least one moderate weakness
	6	Satisfactory	Some strengths but also some moderate weaknesses
Low	7	Fair	Some strengths but with at least one major weakness
	8	Marginal	A few strengths and a few major weaknesses
	9	Poor	Very few strengths and numerous major weaknesses

Summary Paragraph for overall score driving strengths and weaknesses:

The proposal titled "Improving Mental and Physical Health in Rural and Culturally Diverse Populations: Music as a Therapeutic Intervention" aims to address mental health disparities for Indigenous people in a skilled nursing facility using a music-based intervention. The proposal clearly outlines a health equity problem, why this intervention could be particularly useful to this population, and how it can be achieved with the personnel involved. The proposal could be strengthened with more methodological detail in terms of what the intervention will look like, how long it will be implemented and in what way, and the scope of data collection instruments. In addition, there is some hesitation if the relationship with Haven has yet to be formed or any issues with NAU's Office of Native American Initiatives. The tie to SHERC goals are strong and the research team is appropriate for the work being conducted. With some clarifications, this project would be a good fit for the PPP program.

SHERC Pilot Project Program Year 9

SHERC Community Expert Board LOI Review Template

Reviewer: SHERC Community Expert Board Member

PI Name: Katherine Mommaerts and Ariel Roddy

Because of your important work in the communities, we would like to honor your expertise and ask that you to participate in the review process by providing feedback on the questions below related to community priorities and engagement.

This document will be shared with the applicant.

(3) The project's potential to address community priorities and make an impact.

The project titled, "Improving Mental and Physical Health in Rural and Culturally Diverse Populations: Music as a Therapeutic Intervention", is proposed to address community priority by implementing a music-based intervention with Indigenous and non-Indigenous elders housed at Haven Health, a skilled nursing and rehabilitation hospital of Northern Arizona. The objectives didn't include the total number of individuals to receive the intervention nor a current partnership with Indigenous knowledge keepers related to traditional music and songs.

(4) The project's potential for community engagement and/or sharing back results or progress to stakeholders.

The project has the potential for community engagement through the proposed community advisory board that will comprise of Indigenous music experts, music experts, clinicians, and community members who will provide feedback on the intervention components and study procedures. However, the expected outcomes section doesn't include how the intervention will be evaluated to measure impact.

SHERC PPP Letter of Intent Scoring Sheet		
**Reviewer #3**<br>**Proposal PI Name: Mommaerts**		
**This scoring sheet will be shared with the applicant.**		
**Strengths and Weaknesses Summary Template**<br>*Please provide a short summary (1 – 2 sentences) of strengths/weaknesses in each of the LOI instructions/guidelines categories (a – e) below.*		
**LOI instructions/guidelines**	**Strengths**	**Weaknesses**
LOI instructions for a – d below are to*: Briefly describe the nature and scope of the project to include the following:*		
a. Goals of the proposed research	Design and implement music-based interventions (MBIs) to examine the impact that active music therapies (i.e., actively playing music) has on the physical and mental health of a diverse group of individuals living at Haven Health.	
b. Objectives of the research proposed (e.g., to test a stated hypothesis, create a novel design, solve a specific problem, challenge an existing paradigm or clinical practice, address a critical barrier to progress in the field, or develop a new technology)	Evaluate how a culturally-relevant intervention containing music can guide the development of future clinical interventions aimed at promoting overall well-being (i.e., reduce mental and physical health symptoms) and affect health outcomes among Indigenous individuals.	Proposal only states that qualitative data would be used to measure the feasibility and acceptability of the intervention. More detail is needed.<br><br>The stated approach of the study is rooted in understanding that cultural loss and isolation contribute significantly to health disparities. Will a sample be recruited that feels culturally lost and isolated?

	Predict that music enhances working memory, empathy, sense of belonging, social cohesion, self-esteem, connection to community and culture, and increased coping skills to manage physical and mental health conditions.	
c. Summary of the expected outcome, including impact of the results in the research field(s) involved; and	The expected outcomes:<br>1) comprehensive understanding of the feasibility, acceptability, and preliminary impact of a culturally-relevant MBI.<br><br>Quasi-experimental study with pre- and post-test measures. H1: Ps will report an increased use of music as a coping and therapeutic strategy for mental and physical health outcomes. H2: Ps will have a significant reduction in mental health symptoms, including anxiety, depression, and perceived stress, alongside a decrease in physical health symptoms, including pain.	Insufficient justification for all of the proposed effects of an MBI: working memory, empathy, sense of belonging, social cohesion, self-esteem, connection to community and culture, and coping skills. (references could include more related to the proposed effects of an MBI intervention—and if a full proposal is submitted, from relevant disciplinary perspectives).<br><br>Describe Haven Health residents in terms of age, recruitment strategies, and will there be assessment of this being individuals who perceive cultural loss and isolation?
d. How the objectives align with the goals of SHERC and relevance to health equity. (See definitions of health equities and health disparities written in the LOI guidelines document)	Strong.	
e. Investigators		

Is a co-investigator from different area of expertise named? Is the investigative team appropriate?	Strong team; good prospect for R01 on music-based interventions	Given the proposed effects of intervention, I think the team would benefit from music therapist as part of team and/or stress/coping researcher regarding some of the expected outcomes.
**Overall Score (1 – 9): 7**		

Impact	Score	Descriptor	Additional Guidance on Strengths/Weaknesses
High	1	Exceptional	Exceptionally strong with essentially no weaknesses
	2	Outstanding	Extremely strong with negligible weaknesses
	3	Excellent	Very strong with only some minor weaknesses
Medium	4	Very Good	Strong but with numerous minor weaknesses
	5	Good	Strong but with at least one moderate weakness
	6	Satisfactory	Some strengths but also some moderate weaknesses
Low	7	Fair	Some strengths but with at least one major weakness
	8	Marginal	A few strengths and a few major weaknesses
	9	Poor	Very few strengths and numerous major weaknesses

Summary Paragraph for overall score driving strengths and weaknesses: The idea to intervene with a music-based intervention is timely given the NIH's call for R01 grants on this topic. The study approach is stated as being "rooted in understanding that cultural loss and isolation contribute significantly to health disparities" but there is no strong reason to think that residents of Haven Health, an organization whose focus is to provide "care and services that integrate the communal dynamics of the Navajo culture" would be individuals who have no cultural connections. The project design is described as quasi-experimental and multi-method—but no clarification of any specifics (e.g., control group, sample size, duration of intervention, theoretical underpinnings of proposed intervention. The team might benefit from the addition of a music therapist.
